# RAI1 Transcription Factor Activity Is Impaired in Mutants Associated with Smith-Magenis Syndrome

**DOI:** 10.1371/journal.pone.0045155

**Published:** 2012-09-18

**Authors:** Paulina Carmona-Mora, Cesar P. Canales, Lei Cao, Irene C. Perez, Anand K. Srivastava, Juan I. Young, Katherina Walz

**Affiliations:** 1 John P. Hussman Institute for Human Genomics, Miller School of Medicine, University of Miami, Miami, Florida, United States of America; 2 Dr. John T. Macdonald Foundation Department of Human Genetics, Miller School of Medicine, University of Miami, Miami, Florida, United States of America; 3 JC Self Research Institute of Human Genetics, Greenwood Genetic Center, Greenwood, South Carolina, United States of America; 4 Department of Genetics and Biochemistry, Clemson University, Clemson, South Carolina, United States of America; 5 Department of Medicine, Miller School of Medicine, University of Miami, Miami, Florida, United States of America; University of Insubria, Italy

## Abstract

Smith-Magenis Syndrome (SMS) is a complex genomic disorder mostly caused by the haploinsufficiency of the Retinoic Acid Induced 1 gene (*RAI1*), located in the chromosomal region 17p11.2. In a subset of SMS patients, heterozygous mutations in *RAI1* are found. Here we investigate the molecular properties of these mutated forms and their relationship with the resulting phenotype. We compared the clinical phenotype of SMS patients carrying a mutation in *RAI1* coding region either in the N-terminal or the C-terminal half of the protein and no significant differences were found. In order to study the molecular mechanism related to these two groups of RAI1 mutations first we analyzed those mutations that result in the truncated protein corresponding to the N-terminal half of RAI1 finding that they have cytoplasmic localization (in contrast to full length RAI1) and no ability to activate the transcription through an endogenous target: the *BDNF* enhancer. Similar results were found in lymphoblastoid cells derived from a SMS patient carrying *RAI1* c.3103insC, where both mutant and wild type products of RAI1 were detected. The wild type form of RAI1 was found in the chromatin bound and nuclear matrix subcellular fractions while the mutant product was mainly cytoplasmic. In addition, missense mutations at the C-terminal half of RAI1 presented a correct nuclear localization but no activation of the endogenous target. Our results showed for the first time a correlation between RAI1 mutations and abnormal protein function plus they suggest that a reduction of total RAI1 transcription factor activity is at the heart of the SMS clinical presentation.

## Introduction

Smith-Magenis syndrome (SMS, OMIM #182290) is a genomic disorder associated with a microdeletion at chromosome 17 band p11.2 with an estimated prevalence of 1∶15,000–1∶25,000 live births [1], [2]. The clinical characteristics include behavioral problems, sleep abnormalities, intellectual disability, speech delay, growth retardation, brachycephaly, midface hypoplasia, prognathism and hoarse voice, among others [1], [2]. The finding of SMS cases caused by heterozygous mutations within the Retinoic Acid Induced 1 (*RAI1*) sequence, a gene that lies at the common deletion region, indicates that this is the dosage sensitive gene responsible for most of the SMS features [3]–[9].

*RAI1* gene contains six exons, but it is the third exon the one that contains most of the coding sequence and where the majority of *de novo* mutations associated to SMS have been found. RAI1 is a nuclear protein with transcription factor activity [10], of which four isoforms have been described according to the database www.UniProt.org [11]–[13], being isoform 1 the canonical one [14]. While two RAI1 isoforms share high similarity with the canonical, the predicted isoform 4 is the most different one, containing only the N-terminal half of the protein. The defined conserved domains within RAI1 protein include a polyglutamine and two polyserine tracts, a bipartite nuclear localization signal and a zinc finger like plant homeo domain (PHD), which has been found in many chromatin associated proteins [15].

Potocki-Lupski syndrome (PTLS, OMIM #610883) represents the reciprocal duplication, dup(17)(p11.2p11.2), with clinical features including infantile hypotonia, structural cardiovascular anomalies, intellectual disability and features of autistic spectrum disorder [16]. Findings with mouse models for PTLS [17] and the identification of PTLS patients with nonrecurrent duplications containing only *RAI1* in the rearranged interval [18], represent strong evidence to show *RAI1* as the predominant gene responsible for the PTLS phenotype. RAI1 has also been linked with response to neuroleptics in schizophrenia and with the age of onset of spinocerebellar ataxia type 2 [19], [20]. Additionally, *RAI1* has been identified as one of the candidate genes for the susceptibility of autism spectrum disorder [21].

In spite the importance of RAI1 in many complex human traits, there is little knowledge about the protein and the cellular pathways it could be involved, hence more light needs to be shed about its molecular function and its involvement in the pathogenesis of several phenotypes. In the present study, we divided the known human RAI1 mutants into two main groups for their functional analysis: the first one consists of mutations that produce an N-terminal truncated protein approximately half the size of wild type RAI1 isoform 1 and the second group includes missense mutations mapping at the C-terminal half of RAI1. We found that the first group of mutations present an aberrant cytoplasmic subcellular localization and hence the inability to activate the transcription mediated by the *BDNF* enhancer element, an endogenous target of RAI1 [8], while for the second group of mutants analyzed, we found an altered activation of transcription mediated by the *BDNF* enhancer element. When comparing the phenotypes of patients carrying mutations either at the N or C-terminal of RAI1, no significant differences were found to relate the location of the mutations with the clinical features. Altogether these results suggest that the transcription factor activity of RAI1 is at the heart of the SMS pathogenesis.

## Results

### No Differences in Clinical Presentation were Found between SMS Patients Harboring Mutations in the N vs. the C-terminal Portions of RAI1

**Figure 1:**
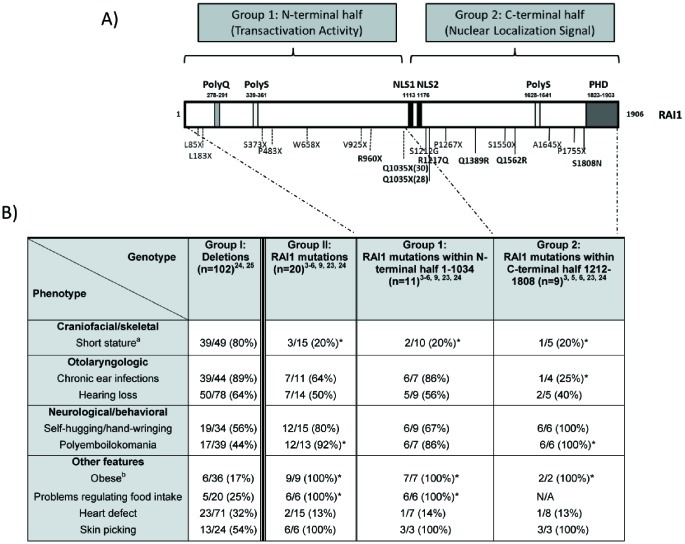
Schematic representation RAI1 protein structure and point mutations associated with the SMS phenotype. A) The structure of isoform 1 of RAI1 protein is schematically represented and the following domains are indicated according to their amino acidic position: polyglutamine tract (PolyQ), two different polyserine tracts (PolyS), a plant homeo domain PHD and bipartite nuclear localization signals, NLSs. Twenty SMS patients harboring a RAI1 point mutation are depicted: two nonsense, five missense and eleven frameshift mutations (including four patients with Q1035X). The two groups of mutations are represented. The mutations analyzed in the present study are shown in bold. **B)** Clinical features previously described as significantly different between SMS patients harboring a common, large, small or atypical deletion or RAI1 point mutation [25] are listed. Four sets of comparisons are shown: **1.** between SMS patients harboring a deletion versus RAI1 point mutation (groups I and II), **2.** between SMS patients harboring a deletion versus RAI1 point mutation in the N-terminal half of the protein (groups I and 1), **3.** between SMS patients harboring a deletion versus RAI1 point mutation in the C-terminal half (groups I and 2), **4.** between SMS patients harboring a RAI1 point mutation in the N-terminal half versus the C-terminal half (groups 1 and 2). The significant differences found in each set of comparisons are represented with an asterisk (* = p<0.05). a: short stature (<5^th^ percentile); b: Obese classification based on Body Mass Index (BMI): BMI >95^th^ percentile for children and adolescents; BMI>30 for adults. N/A = not available.

To date more than twenty patients with SMS have been related to a heterozygous mutation within the *RAI1* coding region [3]–[6], [9], [22]–[24] (figure 1A). We had defined two functional domains in the RAI1 protein, the N-terminal one that has the transactivational activity and the C-terminal that presents the signals for nuclear localization [10]. In order to determine if there is any difference in the clinical presentation among the SMS patients carrying a mutation either at the N-terminal or the C-terminal half of the RAI1 protein, we compared the clinical presentation between patients carrying a common deletion vs. patients whose RAI1 mutation is located between the amino acids 1–1034 of RAI1 (N-terminal portion of the protein), or patients carrying mutations between the amino acids 1212–1906 (within the C-terminal portion of the protein). The clinical presentation of the patients with deletions in 17p11.2 and these two subgroups was revised from the literature and summarized in figure 1B.

Features were first compared between SMS patients harboring a common, large, small or atypical deletion at 17p11.2 versus the cases due to *RAI1* mutations. We analyzed the characteristics that were reported as statistically significant in Edelman *et al.*, 2007 [25]. We found a significant difference in several of the clinical features previously described as short stature, polyemboilokomania, obesity and problems regulating food intake. For hearing loss, self- hugging/hang-wringing, and heart defects (as ventral septal defect, atrial septal defect, tricuspid stenosis, mitral stenosis, tricuspid and mitral regurgitation, aortic stenosis, pulmonary stenosis, mitral valve prolapse, tetralogy of Fallot, and total anomalous pulmonary venous return) we did not find any significant difference may be due to the addition of new reported patients. Then we compared SMS patients harboring *RAI1* mutations within the N-terminal half or at the C-terminal half and no significant differences in clinical presentation were found suggesting a common molecular mechanism for the SMS pathogenesis despite the functional domain of the protein that is affected.

### Abnormal Subcellular Localization is the Main Alteration Related to SMS Associated Mutations Yielding N-terminal Halves of *RAI1*

Nine single nucleotide *de novo* mutations producing a truncation at the first N-terminal half of RAI1 (amino acids 1–1034) have been reported in SMS cases. From these, a 35% correspond to a mutation between the amino acids ∼900–1100 in exon 3 of *RAI1*, that can potentially produce a short form of the RAI1 protein. We studied the pathogenic effects of this group consisting of RAI1 protein products from amino acid 1 to 1034.

We have previously reported the *in vitro* study of RAI1 truncated proteins RAI1 p.R960X (c.2878C>T) and RAI1 p.1035fsX28 (c.3103delC), both associated with the SMS phenotype. For these two mutations, the molecular weights of the resulting proteins were higher than expected and they exhibited cytoplasmic subcellular localization. Interestingly, they were able to retain RAI1 transcription factor activity when assayed as a fusion protein with the GAL4 DNA binding domain (GAL4-BD) [10]. Important is to mention that the GAL4-BD is enough to translocate the fusion protein to the nucleus [26].

**Figure 2:**
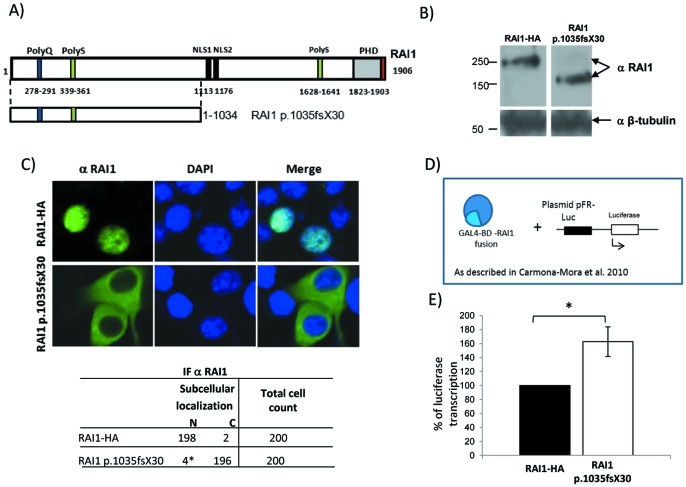
Molecular and cellular evaluation of the p.1035fsX30 truncated protein. A) Schematic representation of the RAI1 protein structure, which includes the polyglutamine tract (PolyQ, blue), polyserine tract (PolyS, green), bipartite nuclear localization signal (NLS, black), and the plant homeo domain (PHD, gray). The HA epitope (red) was added by PCR at the 3′ end of full length cDNA of *RAI1* isoform 1. The p.1035fsX30 truncated protein is represented below the isoform 1 of RAI1. **B)** Neuro-2a cells were transfected either with the clones *RAI1-HA* wild type or *RAI1* c.3103insC (p.1035fsX30). A Western blot using an antibody against RAI1 (α RAI1) was performed to calculate the molecular weight of RAI1 p.1035fsX30. The anti β-tubulin antibody (α β-tubulin) was used as loading control. **C)** The subcellular localization of the truncated protein was assessed by immunofluorescence using anti RAI1 (α RAI1). Neuro-2a cells were transfected with the plasmids coding for *RAI1* wild type and *RAI1* p.1035fsX30. As negative control, untransfected cells are also shown. Nuclei were stained with DAPI. The table indicates the subcellular localization N; nuclear, C; cytoplasmatic, of 200 cells, *: p≤0.05). **D)** Schematic representation of Neuro-2a cells that were co-transfected with *RAI1* wild type or *RAI1* c.3103insC fused with GAL4-BD (pCMV-BD), plus the luciferase reporter plasmid. β-galactosidase activity was used for normalization due to differences in transfection efficiency. **E)** Luciferase expression was utilized as a reporter for transactivation activity of RAI1. The wild type activity was considered as 100%. Values represent mean ± SEM. (RAI1-HA n = 9, RAI1 p.1035fsX30 n = 3; *: p≤0.05).

Due to the importance of the nucleotide 3103, that has been described as a mutation hot spot [9], we have generated a cDNA carrying the mutation c.3103insC (p.1035fsX30) (for details see material and methods), which introduces a premature stop codon in the translation similar to the deletion we have previously analyzed, but with a different set of misincorporated amino acids (figure S1). We evaluated the molecular weight, subcellular localization and transactivational activity of the resulting protein for p.1035fsX30 (figure 2). Wild type or mutant cDNA were transiently transfected into Neuro-2a cells and 48 h post-transfection the cell lysates were run in a 4–20% gradient gel. The predicted molecular weight for the truncated protein p.1035fsX30 was ∼114 kDa. However, the experimental molecular weight of the RAI1 p.1035fsX30 was ∼170 KDa (figure 2) further suggesting that the N-terminal portion of the protein undergo post-translational modifications [10], [27].

Additionally, the theoretical isoelectric point (pI) for the isoform 1 of RAI1 is 9.03 meanwhile the truncated proteins p.1035fsX30 and p.1035fsX28 had a pI of ∼5.5, and the proteins p.R960X and isoform 4 had a theoretical pI of ∼5.23. This may indicate different solubility between RAI1 isoform 1 and the short forms of RAI1 at a given pH, suggesting the possibility of different protein-protein interactions.

The subcellular localization of RAI1 p.1035fsX30 was evaluated by immunofluorescence against a RAI1 N-terminal epitope, in Neuro-2a cells transfected with pAlter-MAX *RAI1* c.3103insC. The resulting p.1035fsX30 protein localizes in the cytoplasm (figure 2). Important is to note that despite the Neuro-2a cells have endogenous Rai1 expression, detected by RT-PCR (data not shown) and Western blot analysis [10], the antibody utilized in this work did not recognize the mouse protein, probably due to a mismatch of 7 amino acids in the recognition sequence.

The transcription factor capacity of RAI1 p.1035fsX30 was evaluated using the same method described previously [10], in which the fusion protein GAL4 DNA binding domain-RAI1 (wild type or the mutated forms) was co-transfected with a plasmid containing the GAL4-binding element upstream of the luciferase coding sequence (figure 2D). RAI1 p.1035fsX30 gave an increment of 165±23.8 in percentage of activation as compared to 100% of the wild type (figure 2E). This result is similar to what was observed for p.1035fsX28 and p.R960X mutants [10], indicating that the N-terminal portion harbors the transcription factor activity domain. In addition, as these truncated proteins showed significantly higher activity than the wild type form we speculate that there may be a regulatory domain of the activity within the C-terminal portion of the RAI1 protein.

### *RAI1* p.1035fsX30 Truncated Protein is Produced in Lymphoblastoid Cells from a Human Patient

**Figure 3:**
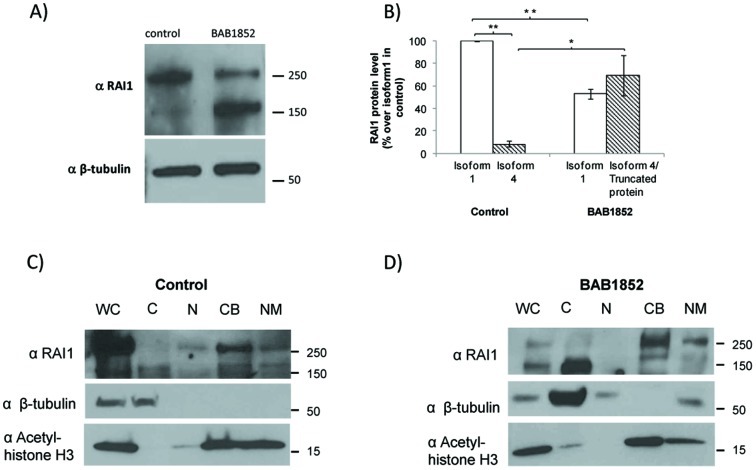
Evaluation of RAI1 expression in lymphoblastoid cells. **A)** Western Blot for RAI1 expression in lymphoblastoid cells from patient BAB1852 and normal control. **B)** A band densitometric analysis of RAI1 isoforms was performed, and the percentages compared to the isoform 1 in cells from the control are shown. The band of ∼170 kDa found in patient cells represents truncated RAI1 isoform 1 and RAI1 isoform 4 proteins as a result of their similar molecular weight. (n = 4 for both samples, * p = 0.02; ** p≤1.78*10^−6^). Subcellular localization of RAI1 in **C)** control or **D)** BAB1852 lymphoblastoid cells. Whole Cell (WC), Cytoplasmic (C), Nuclear soluble (N), Chromatin Bound (CB) and Nuclear Matrix (NM) fractions were obtained from control or BAB1852 lymphoblastoid cells. RAI1 isoform 1 is mainly associated with chromatin and nuclear matrix. The antibodies Acetyl-histone H3 antibody (α Acetyl-histone H3, chromatin bound protein marker) and beta-tubulin antibody (α β-tubulin, cytoplasmic soluble fraction marker) were used as controls of the protocol for cell fractionation.

In order to investigate if there was expression of RAI1 p.1035fsX30 in this SMS patient we utilized lymphoblastoid cells (derived from the BAB1852 patient) that carry a RAI1 c.3103insC allele. Total mRNA was isolated from mutant and control cells. cDNA was produced and Sanger sequenced. We found that both alleles were expressed in the patient cell line (data not shown). Interestingly, a difference in allelic expression (normal allele peaks seem higher than mutant allele peaks) where observed, in agreement with previous reports [9], [24], suggesting that *RAI1* mRNA levels are decreased in lymphoblastoid cells of SMS patients with RAI1 mutations. At the protein level we were able to detect two bands by Western blot analysis utilizing an anti RAI1 antibody, one of 250 kDa and another of ∼170 kDa (figure 3A) for both the control and the mutant samples. However the ratio between both bands was different. In normal control lymphoblastoid cells, the 250 kDa band (corresponding to RAI1 isoform 1) expression level was set to 1 fold and the relative protein level of the 170 kDa band was calculated as 0.08 folds when comparing to isoform 1. In the patient lymphoblastoid cells, RAI1 isoform 1 expression level is similar to the 170 kDa band (0.53 and 0.7 folds respectively) indicating a difference in ratios between both bands in the control and patient sample. In addition, when the abundance of each band is compared between both samples, the expression of the 250 kDa band in the patient corresponds to a 53.0% ±2.58 of the normal control (figure 3B), while the density of the band ∼170 kDa is increased for the patient BAB1852. All these data suggest that the RAI1 p.1035fsX30 is been translated.

Previous reports showed the involvement of RAI1 in the cellular growth and proliferation [7]. We found a significant diminished cellular proliferation and significant increase in population doubling time for patient cells (figure S2). No differences in cell shape, size or viability between both cell samples were observed.

### RAI1 is Mainly Found in the Chromatin Bound Subcellular Fractionation

In order to address the endogenous subcellular localization of wild type RAI1, cytoplasmic extract, nuclear soluble extract, chromatin bound and nuclear matrix fractions were obtained from the control and the mutant samples. The Western blot against a RAI1 (figure 3C **)** clearly shows that the 250 kDa band is mainly found in the “chromatin bound” and “nuclear matrix” fractions. When analyzing the cells from the SMS BAB1852 patient, the 250 kDa band was also mainly found in the “chromatin bound” and “nuclear matrix” fractions while there is a big increment of the 170 kDa protein that was only found in the cytoplasmic fraction (figure 3D).

### Transcription of a Reporter Gene Driven by an Endogenous Target is Impaired in all RAI1 Mutant Forms

**Figure 4:**
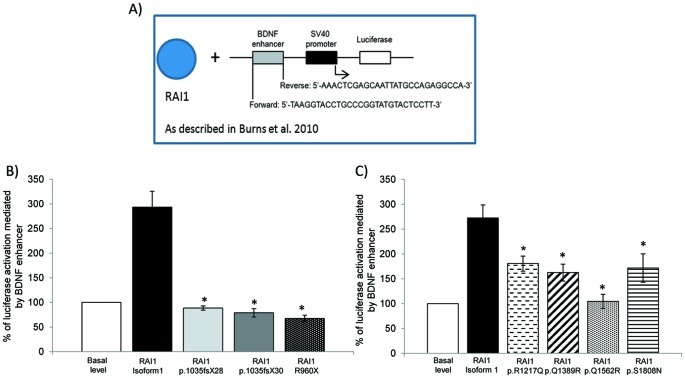
*BDNF* enhancer transactivation activity for two groups of RAI1 mutations. A) Schematic representation of the plasmid construct used to measure the transactivation activity of RAI1 wild type or the mutants forms driven by the *BDNF* enhancer in HEK293T cells. The intronic sequence for *BDNF* gene enhancer region was amplified by PCR and added upstream of the SV40 promoter. **B)** HEK293T cells were co-transfected with a *BDNF* fused luciferase reporter plasmid, a β-galactosidase reporter plasmid, and either *RAI1* isoform 1, p.1035fsX28, p.1035fsX30 or *RAI1* R960X. Forty eight hours post-transfection the reporter proteins were measured from the cell lysates. Activation of the reporter for empty vector along with *BDNF* fused luciferase reporter plasmid and a β-galactosidase reporter plasmid (basal endogenous *BDNF* level) was used for normalization. (p≤0.002). RAI1 isoform 1 n = 9, RAI1 p.1035fsX28 n = 6, RAI1 p.1035fsX30 n = 9, RAI1 R960X n = 6. Values represent mean ± SEM. (* = p<0.05). **C)** The same experiment was performed for group 2 of point mutations mapping at the C-terminal half of RAI1. All the proteins analyzed exhibit impaired activation of the endogenous target *BDNF*. RAI1 isoform 1 n = 3, RAI1 p.R1217Q n = 4, RAI1 p.Q1389R n = 4, RAI1 p.Q1562R n = 6 and RAI1 p.S1808N n = 9. Values represent mean ± SEM. (* = p<0.05.).

To further understand the pathogenic potential of *RAI1* mutations, we assayed the capability of the truncated forms for activating the transcription of a reporter gene driven by an endogenous target: the *BDNF* enhancer sequence previously described by Burns *et al*, 2010 [8]. This intronic sequence was cloned into the pGL3-promoter vector which contains an SV40 promoter upstream of the luciferase coding sequence (pGL3 *BDNF* enhancer) (figure 4A). Forty eight hours after transfection of HEK293T cells with pGL3 *BDNF* enhancer together with the construct pAlter-MAX *RAI1-HA*, there was an activation of 2.93±0.32 folds over the basal expression level of the reporter luciferase (due to the presence of endogenous RAI1 plus the transfection with the empty vector pAlter-Max). However, when cells expressed the RAI1 p.1035fsX28, p.1035fsX30 or p.R960X forms along with the reporter construct no activation was observed (figure 4B). These results reinforce the idea that RAI1 p.1035fsX28, p.1035fsX30 and p.R960X proteins cannot enter the nucleus hence they cannot activate transcription due to incorrect subcellular localization.

*RAI1* missense mutations p.R1217Q, p.Q1389R, p.Q1562R and p.S1808N lie within the C-terminal half of RAI1, but do not map in any domain of the protein described to date. We transfected in HEK293T the pAlter-MAX constructs coding for RAI1 p.R1217Q, p.Q1389R, p.Q1562R and p.S1808N together with the *BDNF* enhancer/reporter plasmid. All mutant proteins were able to induce transcription of the luciferase gene but significantly less than the wild type RAI1 protein (figure 4C).

## Discussion

More than 75% of SMS cases are caused by a common deletion (∼4 Mb) at 17p11.2 [25], [28]–[31]. Interestingly enough, to date more than twenty patients with SMS have been related to a heterozygous mutation within the *RAI1* coding region [3]–[6], [9], [22]–[24]. We have previously defined two main functional domains for RAI1 protein: one at the N-terminal half of RAI1, where the transactivational activity lies, and the second one responsible for the nuclear localization of the protein, encompassing the C-terminal half [10]. We selected the clinical features that showed statistical significant differences between patients harboring a deletion at 17p11.2 and patients carrying *RAI1* mutations [25], and used them to compare patients carrying RAI1 mutations in the N or the C-terminal part of the protein. No significant differences were found. This can be due to a lack of fine clinical description or a result of a common underlying mechanism for all mutations.

The *in vitro* molecular characterization of the mutants associated to SMS that produced N-terminal truncated proteins: p.R960X, p.1035fsX28 and p.1035fs30 showed increased intrinsic transactivation activity when tested as a fusion protein with the GAL4-BD (that can translocate it to the nucleus) compared to the wild type protein, indicating that the transactivation domain resides in the N-terminal region of the protein and suggesting the existence of a regulatory region of the activity within the C-terminal portion of the RAI1 protein. However, abnormal cytoplasmic localization hence inability to activate the transcription mediated by an endogenous target was observed for all the mutants associated to SMS that produced N-terminal truncated proteins.

**Figure 5:**
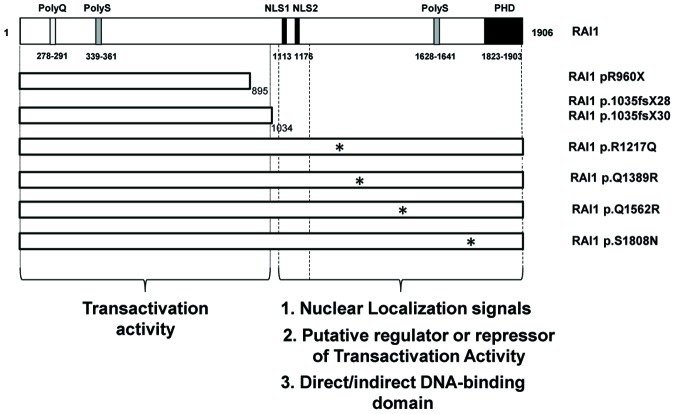
Schematic representation of RAI1 functional domains. The schematic representation of all the mutants analyzed in this study is shown. An asterisk represents the missense mutations. The N and C-terminal halves of the protein are depicted with the description of their functional role.

The mutations RAI1 p.R1217Q, p.Q1389R, p.Q1562R and p.S1808N were previously reported to be located in the nucleus and have the same transactivational activity than wild type RAI1 when assessed as fusion proteins with the GAL4-BD system [10], [23]. But when assessed for their ability to activate the endogenous target, the four missense mutations produced a diminished activation driven by the *BDNF* enhancer when compared to the wild type protein. There two possible explanations for these results: 1. there is a direct or indirect DNA biding site within the C-terminal region that is impaired by the presence of the mutant amino acids, or 2. The regulatory domain that is present in the C-terminal of the protein is negatively affecting the transcription factor activity. All this is summarized in figure 5. In addition, is important to note that this is the first time that mutations in a transcription factor associated with a disease can be adjudicated to either maintenance of transcription activity but retention in cytoplasm versus nuclear translocation but lacking transcription activity.

Interestingly, the N-terminal truncated proteins would have a similar size of the predicted isoform 4 of RAI1 [13]. Our *in vitro* analysis of the N-terminal truncated proteins is confirmatory of what was previously published by Burns *et al.*, 2010 [8] where they generated the predicted isoform 4 of RAI1 that was also found in the cytoplasmic region of the cell. The 170 kDa band that is recognized with the antibody against RAI1 in control lynphoblastoid cells may represent the isoform 4 of RAI1 however; there is a lack of specific antibody against this isoform. Further studies are necessary to really understand if RAI1 isoform 4 is being translated and what its function may be.

RAI1 shares strong homology with SPBP (stromelysin-1 PDGF platelet-derived growth factor-responsive element binding protein), a transcriptional co-regulator involved in the transcriptional activation of the MMP3 (matrix metalloprotease 3) promoter and also modifies the transcriptional activity of several transcription factors or cofactors [32]. Both SPBP and RAI1 were found to bind nucleosomes through their plant homeodomains (PHD). These structural domains are mostly found in nuclear proteins that bind to nucleosomes [33]. It was also demonstrated that PHDs are capable to read the histone modifications [34] and can act as E3 SUMO ligase as well [35], thus corroborating their role in the regulation of transcriptional activity. In addition, SPBP was found to be SUMOylated on its nucleosome binding region [32], while RAI1 has several putative SUMOylation sites (mapping most of them at the N-terminal half). The predicted presence of these SUMO sites, that play an important role in the interaction of a protein with other macromolecules within RAI1 sequence, plus that evidence that PHDs may mediate SUMOylation, further suggest how both N and C-terminal domains are important to modulate RAI1 interactions and its effect in gene regulation.

We have shown the characterization on two groups of RAI1 mutations classified by their localization in the domains previously defined for the protein: the N-terminal half (amino acids 1–1034) where the transactivational activity lies, and the C-terminal half responsible for nuclear localization, the regulation of the transcriptional activity and direct or indirect biding to DNA (see figure 5 for a summary). All RAI1 mutations tested in this work showed impaired transcriptional activation of a reporter gene driven by an endogenous RAI1 target sequence. Therefore, although the molecular aspect that contributes to the pathogenesis of SMS is different for different mutants, the final outcome results in impaired transcriptional activity of RAI1, which seems to be the most critical feature of the protein known up to date.

## Methods

### Plasmid Constructs

The complete *RAI1* cDNA and the mutations *RAI1* c.2878C>T and c.3103delC were previously generated [10]. The single base change *RAI1* c.3103insC was obtained from cDNA isolated from a lymphoblastoid cell line obtained from patient BAB1852 [4] (kindly donated by Dr. James Lupski). A small fragment of *RAI1* cDNA containing the base 3103 was amplified by PCR with the following primers, forward: 5′-GTGGGCTGCTGCAGTG-3′ and reverse: 5′-GGTGGCACGGCAGTTG-3′. The product was cloned into pGEM-T Easy vector (Promega Corporation) and then the clones were verified by DNA sequencing. The fragment containing the mutation was subcloned into full length *RAI1* cDNA with the enzymes *Age*I and *Afe*I.

For expression analysis, the cDNAs of *RAI1* wild type and mutant forms were subcloned into pALTER-MAX vector (Promega Corporation).

Accession numbers: Human RAI1 NCBI Nucleotide RefSeq NM_030665. Isoform 1 Uniprot: Q7Z5J4; isoform 4, Uniprot Q7S5J4-4.

### Cell Culture

Neuro-2a (ATCC# CCL-131) and HEK-293 cells (ATCC# CRL-1573) were grown in Dulbecco's Modified Eagle Medium (Cellgro, Mediatech, Inc) supplemented with 10% fetal bovine serum (Gibco, Life Technologies), penicillin (100 U/mL) and streptomycin (100 µg/mL) (Gibco, Life Technologies) at 37°C with 5% CO_2_ until 95% confluence was attained.

Lymphoblastoid cell lines were obtained from patient BAB1852 and from a control donor. The cells were grown in suspension in RPMI 1640 medium (Cellgro, Mediatech, Inc) supplemented with 10% fetal bovine serum, 100 U/mL penicillin, and 100 µg/mL streptomycin and maintained at 37°C with 5% CO_2_.

### RNA Extraction and RT-PCR

Total RNA was isolated from lymphoblastoid cell lines with TRIzol Reagent (Ambion, Life Technologies) according to manufacturer’s instructions. To avoid genomic DNA contamination, RNA samples were treated with rDNAse I (DNA-free kit; Ambion, Life Technologies). Then cDNA was synthesized using ImProm-II Reverse Transcription System (Promega Corporation).

### Western Blot Analyses and Immunofluorescence

To study the expression of the proteins, Neuro-2a cells were transfected with the plasmids pALTER-MAX *RAI1-HA* wild type and *RAI1* c.3103insC. All transfections were performed using Lipofectamine 2000 (Gibco, Life Technologies) according to manufacturer’s protocol.

For Western blots of transfected cells, Neuro-2a cells were lysed 48 h after transfection in 100 µL of protein extraction and loading buffer (2% SDS, 2 M Urea, 10% Glycerol, 10 mM Tris pH 6.8, 0.002% Bromophenol Blue and 10 mM DTT) plus 1∶200 protease inhibitor cocktail (SIGMA). After incubation at 4°C for 15 minutes, the samples were homogenized by passing 20 times through a syringe and heated to 95°C for 5 minutes. 25 µL of each lysate was loaded onto 4–20% gradient SDS-polyacrylamide gel and transferred to a 0.2 µm poly-vinylidene fluoride (PVDF, Bio-Rad Laboratories) membrane. Immunodetection was performed using rabbit anti human RAI1 polyclonal antibody (1∶1000, Abcam) whose epitope locates at the amino terminal of RAI1, and rabbit anti β-tubulin (1∶1000, sc-9104 Santa Cruz Biotechnology, Inc). Additionally, an Acetyl-histone H3 antibody (1∶10,000, Upstate) was used as control for immunodetection of cell fractionation samples. The results were visualized by chemiluminiscence. Protein expression levels were first quantified using Quantity One Software (Bio-rad Laboratories). RAI1 isoform 1 (250 kDa band) expression level was set to one in each individual Western blot. Statistical analysis was calculated with two-tailed Student’s t-test and a P-value ≤0.05 was considered significant.

For immunofluorescence in Neuro-2a cells, 24 h after transfection with the pALTER-MAX constructs, the cells were fixed with 4% paraformaldehyde and permeabilized with 0.1% Triton X-100 in PBS. Subcellular localization of RAI1 was detected using rabbit anti human RAI1 polyclonal antibody (1∶1000, Abcam). A secondary antibody conjugated to Alexa fluor 488 (1∶1000, Molecular Probes, Life Technologies) was used. Cells were stained with 4',6-diamidino-2-phenylindole, dihydrochloride (DAPI) and mounted with fluorescent mounting medium (Dako).

### Transcriptional Activity Assays

In order to evaluate the transactivation activity of RAI1, transient transfections in Neuro-2a cells were performed in 35 mm plates with Lipofectamine 2000 (Gibco, Life Technologies). The amounts of plasmidial DNA used were according to manufacturer’s protocol. GAL4-BD fusions of human *RAI1* wild type and *RAI1* c.3103insC (pCMV-BD vector, catalog number 211342, Agilent Technologies) were co-transfected with the luciferase reporter plasmid pFR-Luc (catalog number 219050, Agilent Technologies). For normalizing the results in case of transfection efficiency variations, the vector pSV-β-Galactosidase (Promega Corporation) was also co-transfected for expression of β-Galactosidase. After 48 h post-transfection, the cells were lysed and the luciferase activity was measured with Luciferase Assay Kit (Agilent Technologies) according to manufacturer’s instructions. The Relative Lights Units (RLUs) of luciferase were measured in duplicate in a luminometer (Turner BioSystems 20/20n, Promega Corporation). The β-Galactosidase activity was measured in duplicates with the β-Galactosidase Assay kit (microassay protocol, Agilent Technologies).

For assessing the ability of RAI1 and its mutant forms for activating the transcription through the *BDNF* enhancer, we amplified it by PCR from human genomic DNA as template. The primers: forward, 5′-TAAGGTACCTGCCCGGTATGTACTCCTT-3′ and reverse, 5′-AAACTCGAGCAATTATGCCAGAGGCCA-3′ were utilized. The PCR product was digested with *Kpn*I and *Xho*I and ligated into a pGL3-promoter vector (Promega Corporation) to obtain the pGL3 *BDNF* enhancer plasmid. The plasmids pGL3 *BDNF* enhancer, pSV-β-Galactosidase (Promega Corporation) and pAlter-MAX *RAI1-HA* (or pAlter-MAX *RAI1* mutations) were co-transfected in HEK293T cells. 48 h post-transfection, cells were lysed for measuring in duplicate luciferase and β-Galactosidase activities as described previously. Cells co-transfected with pGL3 *BDNF* enhancer, pSV-β-Galactosidase and empty pAlter-MAX vector were used to normalize the data to basal *BDNF* activation levels.

### Cell Fractionation

Cell fractionation was essentially done according to a previously published protocol [36]. 10^7^ lymphoblastoid cells were washed with cold PBS and re-suspended in 200 µL of cytoskeleton buffer (10 mM Pipes pH 6.8, 100 mM NaCl, 300 mM sucrose, 3 mM MgCl_2_, 1 mM EGTA and 0.5% Triton X-100) and incubated in a rotator for 5 minutes at 4°C. Soluble cytoplasmic fraction was isolated by centrifugation at 600×g for 5 minutes. The pellet was incubated for 5 minutes in 200 µL of nuclear soluble buffer (10 mM Tris HCl pH 7.4, 10 mM NaCl, 3 mM MgCl_2_, 1% Tween 40 and 0.5% sodium deoxycolate). Insoluble cytoskeleton and nuclear soluble proteins were isolated by centrifugation at 600×g for 5 minutes. Chromatin DNA was removed with 40 U of RNase-free DNase I (Epicentre) at room temperature for 20 minutes in digestion buffer (10 mM Pipes pH 6.8, 50 mM NaCl, 300 mM sucrose, 3 mM MgCl_2_, 1 mM EGTA, and 0.5% Triton X-100). Ammonium sulfate was added to a final concentration of 0.25 M and the sample was incubated at 4°C for 5 minutes and then pelleted. The supernatant was removed in a new tube as chromatin bound fraction. 100 µL of SDS buffer (25 mM Tris-Cl pH 7.4, 25 mM Sodium Citrate, 2% SDS, 5 mM CaCl_2_, 0.5 mM DTT) was added to the pellet and boiled for 5 minutes to dissolve the nuclear matrix (NM). 1∶100 protease inhibitor cocktail was added in all lysis buffers. 10% of each fraction was used for loading onto SDS-polyacrylamide gel.

### Statistical Analysis

Statistical analysis of comparisons made between clinical presentations of SMS patients was performed using GraphPad software. Fisher’s exact test was calculated using a two tailed P-value significance level of ≤0.05. For the experimental procedures involving numerical values; mean values and standard deviations were calculated with Excel software (Microsoft). The results are given as mean ± standard error of the mean (SEM). Error bars represent standard errors of the mean. Statistically significant differences were evaluated using Student’s t-test; a p≤0.05 was considered as statistically significant.

## Supporting Information

Figure S1 **Short forms of RAI1 containing the N-terminal half of the protein. A)** Representation of the protein structures for RAI1 isoforms 1 and 4, and the proteins RAI1 p.R960X, p.1035fsX28 and p.1035fsX30. The depicted domains include the polyglutamine tract (PolyQ, blue), polyserine tract (PolyS, green), bipartite nuclear localization signal (NLS, black), and the plant homeo domain (PHD, gray). RAI1 isoform 1 is composed by 1906 amino acids and RAI1 splice variant isoform 4 is 966 amino acids. The lengths of the truncated proteins associated with SMS are also shown. **B)** The sequence similarities at the end of the short forms of RAI1 are shown, as well as the misincorporation of amino acids in the mutants RAI1 p.1035fsX28 and p.1035fsX30.
(TIF)

Figure S2 **Effect of** ***RAI1*** **dosage on cell proliferation. A)** Control and BAB1852 patients cells were grown with an initial cell concentration of 40,000 cells/mL in triplicate for 72 hours, cell concentrations were determined with a hemocytometer at 24 h, 48 h and 72 h. * = p<0.05 (Student’s t-test) (n = 3). **B)** Cell population doubling time of control and BAB1852 lymphoblastoid cell lines are shown. Control cells have a doubling time of 48.19 hours, while BAB1852 lymphoblastoid cell line has a prolonged population doubling time of 70.04 hours. * = p<0.05 (Student’s t-test) (n = 3).
(TIF)

## References

[1] Greenberg F, Lewis RA, Potocki L, Glaze D, Parke J, et al (1996) Multi-disciplinary clinical study of Smith-Magenis syndrome (deletion 17p11.2). Am J Med Genet 62: 247–254. 8882782 10.1002/(SICI)1096-8628(19960329)62:3<247::AID-AJMG9>3.0.CO;2-Q

[2] Laje G, Morse R, Richter W, Ball J, Pao M, et al (2010) Autism spectrum features in Smith-Magenis syndrome. Am J Med Genet C Semin Med Genet 154C: 456–462. 20981775 10.1002/ajmg.c.30275 PMC2967410

[3] Slager RE, Newton TL, Vlangos CN, Finucane B, Elsea SH (2003) Mutations in RAI1 associated with Smith-Magenis syndrome. Nat Genet 33: 466–468. 12652298 10.1038/ng1126

[4] Bi W, Saifi GM, Shaw CJ, Walz K, Fonseca P, et al (2004) Mutations of RAI1, a PHD-containing protein, in nondeletion patients with Smith-Magenis syndrome. Hum Genet 115: 515–524. 15565467 10.1007/s00439-004-1187-6

[5] Girirajan S, Elsas LJ 2nd, Devriendt K, Elsea SH (2005) RAI1 variations in Smith-Magenis syndrome patients without 17p11.2 deletions. J Med Genet 42: 820–828. 15788730 10.1136/jmg.2005.031211 PMC1735950

[6] Bi W, Saifi GM, Girirajan S, Shi X, Szomju B, et al (2006) RAI1 point mutations, CAG repeat variation, and SNP analysis in non-deletion Smith-Magenis syndrome. Am J Med Genet A 140: 2454–2463. 17041942 10.1002/ajmg.a.31510

[7] Girirajan S, Truong HT, Blanchard CL, Elsea SH (2009) A functional network module for Smith-Magenis syndrome. Clin Genet 75: 364–374. 19236431 10.1111/j.1399-0004.2008.01135.x PMC13600801

[8] Burns B, Schmidt K, Williams SR, Kim S, Girirajan S, et al (2010) Rai1 haploinsufficiency causes reduced Bdnf expression resulting in hyperphagia, obesity and altered fat distribution in mice and humans with no evidence of metabolic syndrome. Hum Mol Genet 19: 4026–4042. 20663924 10.1093/hmg/ddq317 PMC7714048

[9] Truong HT, Dudding T, Blanchard CL, Elsea SH (2010) Frameshift mutation hotspot identified in Smith-Magenis syndrome: case report and review of literature. BMC Med Genet 11: 142. 20932317 10.1186/1471-2350-11-142 PMC2964533

[10] Carmona-Mora P, Encina CA, Canales CP, Cao L, Molina J, et al (2010) Functional and cellular characterization of human Retinoic Acid Induced 1 (RAI1) mutations associated with Smith-Magenis Syndrome. BMC Mol Biol 11: 63. 20738874 10.1186/1471-2199-11-63 PMC2939504

[11] Seranski P, Hoff C, Radelof U, Hennig S, Reinhardt R, et al (2001) RAI1 is a novel polyglutamine encoding gene that is deleted in Smith-Magenis syndrome patients. Gene 270: 69–76. 11404004 10.1016/s0378-1119(01)00415-2

[12] Nagase T, Nakayama M, Nakajima D, Kikuno R, Ohara O (2001) Prediction of the coding sequences of unidentified human genes. XX. The complete sequences of 100 new cDNA clones from brain which code for large proteins in vitro. DNA Res 8: 85–95. 11347906 10.1093/dnares/8.2.85

[13] Gerhard DS, Wagner L, Feingold EA, Shenmen CM, Grouse LH, et al (2004) The status, quality, and expansion of the NIH full-length cDNA project: the Mammalian Gene Collection (MGC). Genome Res 14: 2121–2127. 15489334 10.1101/gr.2596504 PMC528928

[14] Toulouse A, Rochefort D, Roussel J, Joober R, Rouleau GA (2003) Molecular cloning and characterization of human RAI1, a gene associated with schizophrenia. Genomics 82: 162–171. 12837267 10.1016/s0888-7543(03)00101-0

[15] Sutherland HG, Mumford GK, Newton K, Ford LV, Farrall R, et al (2001) Large-scale identification of mammalian proteins localized to nuclear sub-compartments. Hum Mol Genet 10: 1995–2011. 11555636 10.1093/hmg/10.18.1995

[16] Potocki L, Bi W, Treadwell-Deering D, Carvalho CM, Eifert A, et al (2007) Characterization of Potocki-Lupski syndrome (dup(17)(p11.2p11.2)) and delineation of a dosage-sensitive critical interval that can convey an autism phenotype. Am J Hum Genet 80: 633–649. 17357070 10.1086/512864 PMC1852712

[17] Walz K, Paylor R, Yan J, Bi W, Lupski JR (2006) Rai1 duplication causes physical and behavioral phenotypes in a mouse model of dup(17)(p11.2p11.2). J Clin Invest 116: 3035–3041. 17024248 10.1172/JCI28953 PMC1590269

[18] Zhang F, Potocki L, Sampson JB, Liu P, Sanchez-Valle A, et al (2010) Identification of uncommon recurrent Potocki-Lupski syndrome-associated duplications and the distribution of rearrangement types and mechanisms in PTLS. Am J Hum Genet 86: 462–470. 20188345 10.1016/j.ajhg.2010.02.001 PMC2833368

[19] Joober R, Benkelfat C, Toulouse A, Lafreniere RG, Lal S, et al (1999) Analysis of 14 CAG repeat-containing genes in schizophrenia. Am J Med Genet 88: 694–699. 10581491

[20] Hayes S, Turecki G, Brisebois K, Lopes-Cendes I, Gaspar C, et al (2000) CAG repeat length in RAI1 is associated with age at onset variability in spinocerebellar ataxia type 2 (SCA2). Hum Mol Genet 9: 1753–1758. 10915763 10.1093/hmg/9.12.1753

[21] van der Zwaag B, Franke L, Poot M, Hochstenbach R, Spierenburg HA, et al (2009) Gene-network analysis identifies susceptibility genes related to glycobiology in autism. PLoS One 4: e5324. 19492091 10.1371/journal.pone.0005324 PMC2683930

[22] Girirajan S, Vlangos CN, Szomju BB, Edelman E, Trevors CD, et al (2006) Genotype-phenotype correlation in Smith-Magenis syndrome: evidence that multiple genes in 17p11.2 contribute to the clinical spectrum. Genet Med 8: 417–427. 16845274 10.1097/01.gim.0000228215.32110.89

[23] Vieira GH, Rodriguez JD, Carmona-Mora P, Cao L, Gamba BF, et al (2012) Detection of classical 17p11.2 deletions, an atypical deletion and RAI1 alterations in patients with features suggestive of Smith-Magenis syndrome. Eur J Hum Genet 20: 148–154. 21897445 10.1038/ejhg.2011.167 PMC3260931

[24] Vilboux T, Ciccone C, Blancato JK, Cox GF, Deshpande C, et al (2011) Molecular analysis of the Retinoic Acid Induced 1 gene (RAI1) in patients with suspected Smith-Magenis syndrome without the 17p11.2 deletion. PLoS One 6: e22861. 21857958 10.1371/journal.pone.0022861 PMC3152558

[25] Edelman EA, Girirajan S, Finucane B, Patel PI, Lupski JR, et al (2007) Gender, genotype, and phenotype differences in Smith-Magenis syndrome: a meta-analysis of 105 cases. Clin Genet 71: 540–550. 17539903 10.1111/j.1399-0004.2007.00815.x

[26] Silver PA, Keegan LP, Ptashne M (1984) Amino terminus of the yeast GAL4 gene product is sufficient for nuclear localization. Proc Natl Acad Sci U S A 81: 5951–5955. 6091123 10.1073/pnas.81.19.5951 PMC391836

[27] Carmona-Mora P, Walz K (2010) Retinoic Acid Induced 1, RAI1: A Dosage Sensitive Gene Related to Neurobehavioral Alterations Including Autistic Behavior. Curr Genomics 11: 607–617. 21629438 10.2174/138920210793360952 PMC3078685

[28] Juyal RC, Figuera LE, Hauge X, Elsea SH, Lupski JR, et al (1996) Molecular analyses of 17p11.2 deletions in 62 Smith-Magenis syndrome patients. Am J Hum Genet 58: 998–1007. 8651284 PMC1914618

[29] Potocki L, Shaw CJ, Stankiewicz P, Lupski JR (2003) Variability in clinical phenotype despite common chromosomal deletion in Smith-Magenis syndrome [del(17)(p11.2p11.2)]. Genet Med 5: 430–434. 14614393 10.1097/01.gim.0000095625.14160.ab

[30] Vlangos CN, Yim DK, Elsea SH (2003) Refinement of the Smith-Magenis syndrome critical region to approximately 950kb and assessment of 17p11.2 deletions. Are all deletions created equally? Mol Genet Metab 79: 134–141. 12809645 10.1016/s1096-7192(03)00048-9

[31] Gropman AL, Elsea S, Duncan WC Jr, Smith AC (2007) New developments in Smith-Magenis syndrome (del 17p11.2). Curr Opin Neurol 20: 125–134. 17351481 10.1097/WCO.0b013e3280895dba

[32] Darvekar S, Johnsen SS, Eriksen AB, Johansen T, Sjottem E (2012) Identification of two independent nucleosome-binding domains in the transcriptional co-activator SPBP. Biochem J 442: 65–75. 22081970 10.1042/BJ20111230

[33] Bienz M (2006) The PHD finger, a nuclear protein-interaction domain. Trends Biochem Sci 31: 35–40. 16297627 10.1016/j.tibs.2005.11.001

[34] Mellor J (2006) It takes a PHD to read the histone code. Cell 126: 22–24. 16839870 10.1016/j.cell.2006.06.028

[35] Peng J, Wysocka J (2008) It takes a PHD to SUMO. Trends Biochem Sci 33: 191–194. 18406149 10.1016/j.tibs.2008.02.003

[36] He DC, Nickerson JA, Penman S (1990) Core filaments of the nuclear matrix. J Cell Biol 110: 569–580. 2307700 10.1083/jcb.110.3.569 PMC2116036

