# Supplementary figures and images for: RAI1 Transcription Factor Activity Is Impaired in Mutants Associated with Smith-Magenis Syndrome
Source: PLoS One. 2012 Sep 18;7(9):e45155. doi: 10.1371/journal.pone.0045155 (PMC3445574; doi:10.1371/journal.pone.0045155)

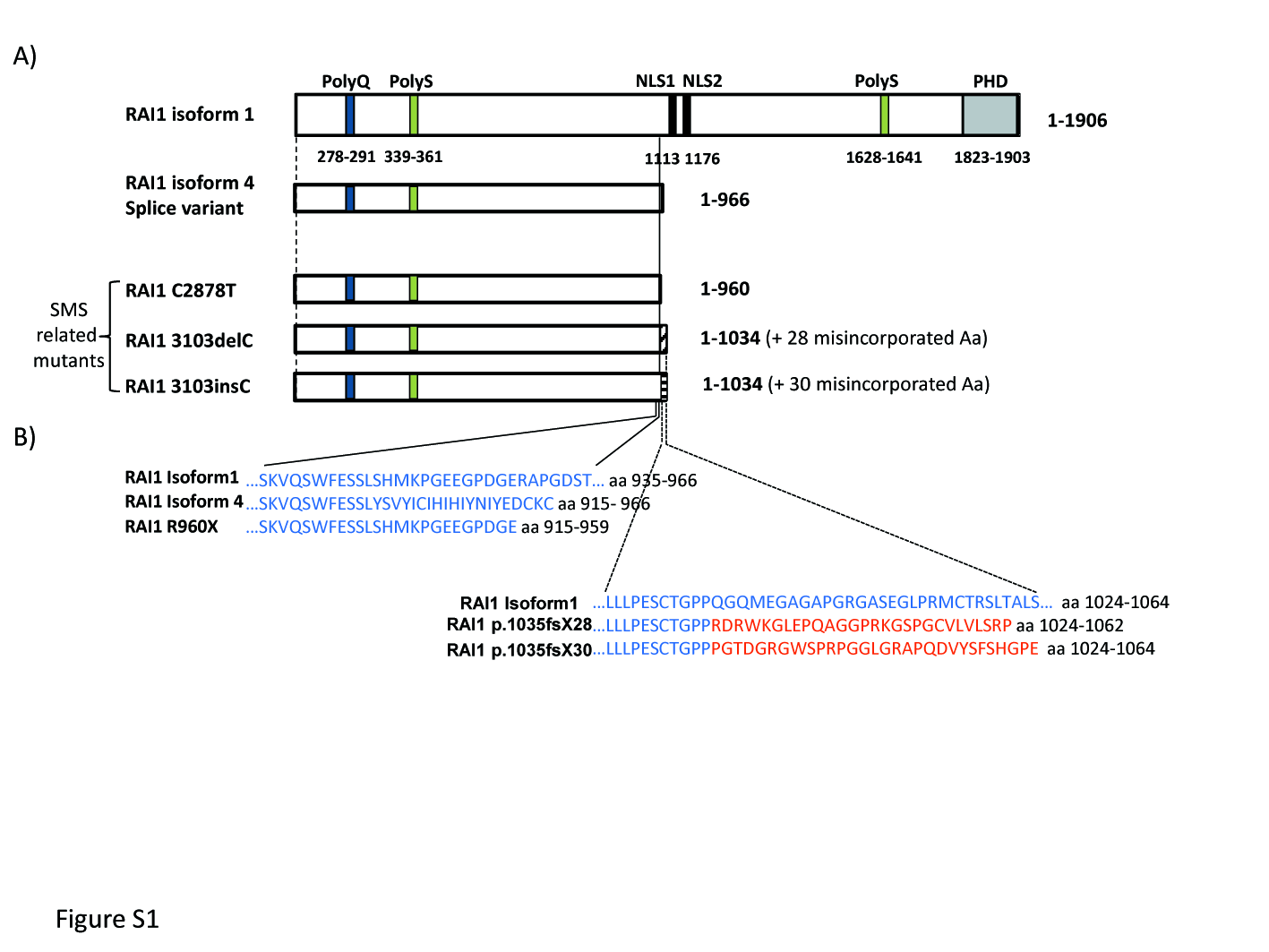

Supplement: Figure S1 — Short forms of RAI1 containing the N-terminal half of the protein. A) Representation of the protein structures for RAI1 isoforms 1 and 4, and the proteins RAI1 p.R960X, p.1035fsX28 and p.1035fsX30. The depicted domains include the polyglutamine tract (PolyQ, blue), polyserine tract (PolyS, green), bipartite nuclear localization signal (NLS, black), and the plant homeo domain (PHD, gray). RAI1 isoform 1 is composed by 1906 amino acids and RAI1 splice variant isoform 4 is 966 amino acids. The lengths of the truncated proteins associated with SMS are also shown. B) The sequence similarities at the end of the short forms of RAI1 are shown, as well as the misincorporation of amino acids in the mutants RAI1 p.1035fsX28 and p.1035fsX30. (TIF) [file pone.0045155.s001.tif]

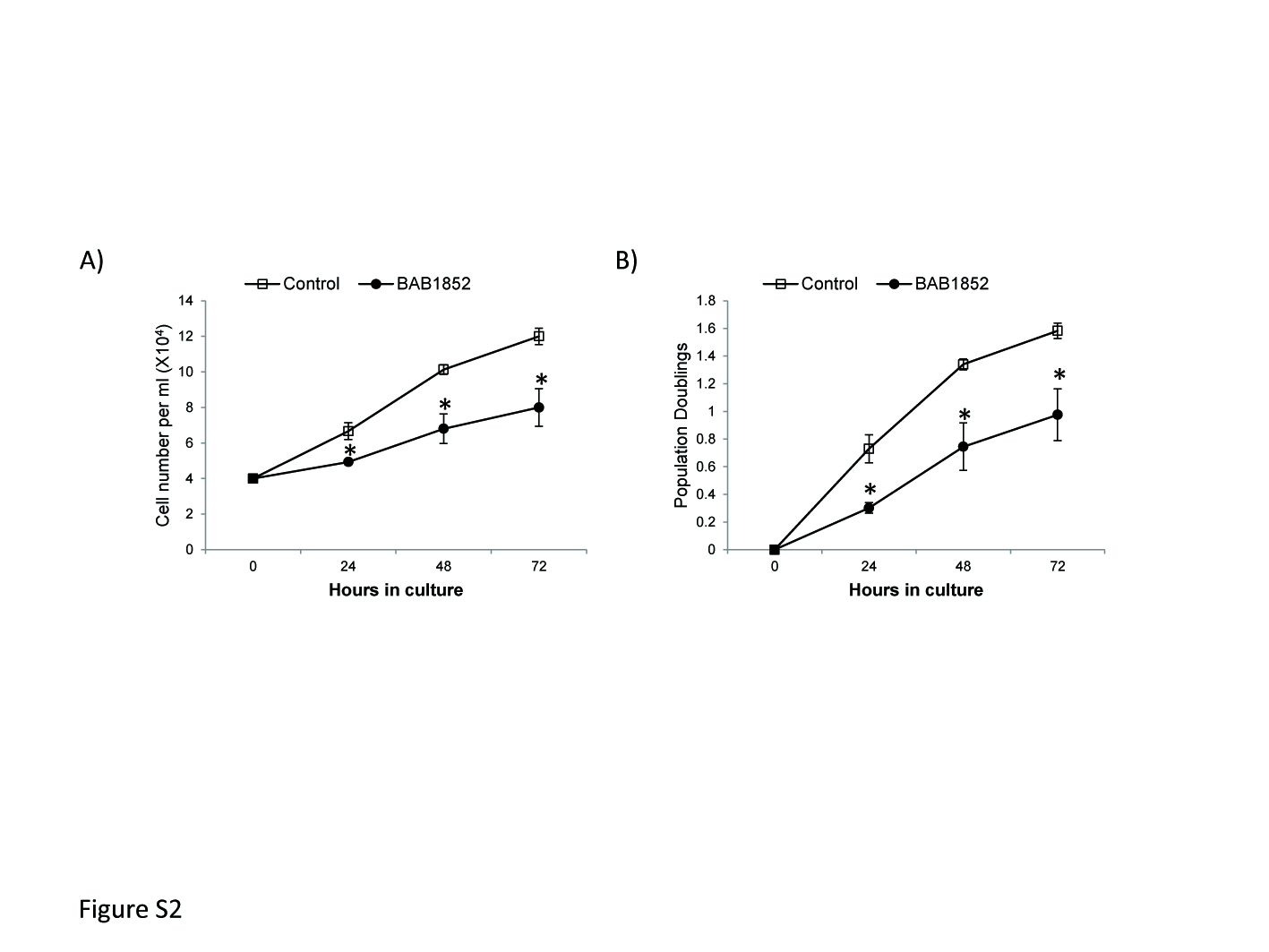

Supplement: Figure S2 — Effect of RAI1 dosage on cell proliferation. A) Control and BAB1852 patients cells were grown with an initial cell concentration of 40,000 cells/mL in triplicate for 72 hours, cell concentrations were determined with a hemocytometer at 24 h, 48 h and 72 h. * = p<0.05 (Student’s t-test) (n = 3). B) Cell population doubling time of control and BAB1852 lymphoblastoid cell lines are shown. Control cells have a doubling time of 48.19 hours, while BAB1852 lymphoblastoid cell line has a prolonged population doubling time of 70.04 hours. * = p<0.05 (Student’s t-test) (n = 3). (TIF) [file pone.0045155.s002.tif]
